# Detection, virus isolation, and phylogenetic analysis of PRRSV2 in China from 2022 to 2025

**DOI:** 10.1186/s40813-026-00531-3

**Published:** 2026-07-11

**Authors:** Hao Yang, Wenbo Song, Desheng Chu, Zechuan Li, Zhanwei Zhu, Rui Xie, Lin Hua, Huanchun Chen, Bin Wu, Zhong Peng

**Affiliations:** 1 National Key Laboratory of Agricultural Microbiology, College of Veterinary Medicine, Huazhong Agricultural University, Wuhan, 430070 China; 2 Hubei Hongshan Laboratory, Wuhan, 430070 China; 3 Frontiers Center for Animal Breeding and Sustainable Production, The Cooperative Innovation Center for Sustainable Pig Production, Wuhan, 430070 China; 4 Animal disease diagnostic Center, Keqian Biology Co., Ltd., Wuhan, 430206 China; 5 COFCO Joycome Foods Limited, Beijing, 100000 China; 6 Hainan Research Institute of Huazhong Agricultural University, Sanya, 572000 China

**Keywords:** PRRSV2, Detection of clinical samples, Virus isolation, Whole genome sequencing, Phylogenetic analysis

## Abstract

**Background:**

Porcine reproductive and respiratory syndrome (PRRS) is one of the most important diseases threatening the global pig industry. It has been reported that PRRSV predominantly circulating in clinical practice in China belongs to the genotype 2 (PRRSV2), comprising four lineages: lineages 1, 3, 5, and 8. Among these, lineage 1 strains are further divided into sublineage 1.5 (NADC34-like strains) and sublineage 1.8 (NADC30-like strains).To further understand the prevalence and variation characteristics of PRRSV2 in China, this study conducted detection, virus isolation, and genetic evolution analysis of PRRSV2 from 2022 to 2025.

**Results:**

This study employed qRT-PCR to detect PRRSV2 in 23,962 samples collected nationwide from 2022 to 2025, with an average positive detection rate of 33.29%. Phylogenetic analysis based on the ORF5 gene revealed that sublineage 1.8 PRRSV was predominant in China, followed by sublineage 1.5 and lineage 8. A total of 389 PRRSV2 strains were isolated using porcine alveolar macrophage (PAM) cells, of which 59 strains could proliferate in Marc-145 cells. Different amino acid variations, insertions, and deletions were identified in the ORF5 and Nsp2 genes of the 389 PRRSV2 strains. Whole genome sequencing was performed on 79 PRRSV strains, and 93.67% (74 strains) exhibited inter-lineage recombination.

**Conclusion:**

This study demonstrates the positive detection rate and predominant strain types of PRRSV2 in China. Sequencing results of isolated PRRSV strains reveals amino acid variations and recombination in the ORF5, Nsp2, and whole genomes. Overall, this study provides data support for the molecular epidemiology of PRRSV2 in China.

## Background

Porcine reproductive and respiratory syndrome (PRRS) is one of the most important diseases in the swine industry, causing substantial economic losses to the global swine industry [1, 2]. Porcine reproductive and respiratory syndrome virus (PRRSV) belongs to the family *Arteriviridae*, genus *Arterivirus*, and is an enveloped, single-stranded positive-sense RNA virus [3]. GP5 is one of the most variable structural proteins in PRRSV. In addition to amino acid variations, studies have shown that there may be deletions of 1–2 amino acids at positions 32–34 [4–6]. The GP5 protein is the primary antigen inducing neutralizing antibodies, containing two B-cell epitopes: the non-neutralizing epitope A and the neutralizing epitope B [4, 7]. Nsp2 exhibits the highest variability in the PRRSV genome, with amino acid homologies of 66%-70% within the same genotype and only approximately 30% between different genotypes [8, 9]. Compared with VR-2332, Nsp2 of different PRRSV types exhibits characteristic deletions [10–14].

PRRSV strains are classified into two genotypes: PRRSV1 and PRRSV2, with prototype strains Lelystad Virus isolated in the Netherlands in 1991 and VR-2332 isolated in the United States in 1992, respectively [15, 16]. Current molecular epidemiological studies have demonstrated that PRRSV2 is the predominant circulating strain in China, mainly comprising lineage 1 (NADC34-like and NADC30-like), lineage 3 (QYYZ-like), lineage 5 (VR-2332-like), and lineage 8 (CH-1a-like and JXA1-like) [17, 18].

Before 2013, the predominant circulating strains in China were lineage 8 [19, 20]. In 2013, sublineage 1.8 was introduced into China and rapidly became the predominant circulating strain in the country [21–23]. In 2017, sublineage 1.5 was introduced into China, and subsequently, the emergence of this strain has been increasingly reported in provinces across the country [24–26]. In recent years, sublineage 1.5 has reached a certain proportion in some regions, demonstrating the potential to become a predominant circulating strain [27].

The current prevalence of PRRSV in China is relatively complex, and the distribution and genomic characteristics of various lineages in clinical practice remain to be further clarified. Therefore, this study conducted clinical sample detection and phylogenetic analysis of the ORF5 gene for PRRSV2 in China from 2022 to 2025, to comprehensively understand the prevalence of PRRSV2 in China. Through virus isolation, amino acid variation analysis, and recombination analysis, the recombination and variation of circulating strains in China were elucidated.

## Materials and methods

### Sample collection and processing

From 2022 to 2025, a total of 23,962 samples were collected from pigs with abortion or respiratory symptoms on farms across 30 provincial-level administrative regions in China. Sample types included tissues (lung, spleen, lymph node, and tonsil, etc.), blood, and swabs. Tissue samples were cut into small pieces and homogenized with PBS using a tissue grinder. Swab samples were added to PBS and vortexed uniformly. Tissue and swab samples were centrifuged at 12,000 r/min for 5 min. Blood samples were left untreated. All samples were stored at -80 °C. All samples in this study were provided by farms.

### Primer design and synthesis

Primers targeting the PRRSV ORF5 gene were designed and synthesized: ORF5-F: 5’-GGCGACCGTTTTAGCCTGTCTT-3’, ORF5-R: 5’-ATCATTATTGGCGTGTAGGTG-3’ [28]. The thermal cycling conditions for PCR were as follows: reverse transcription at 50 °C for 30 min, initial denaturation at 94 °C for 2 min, followed by 35 cycles of denaturation at 94 °C for 30 s, annealing at 54 °C for 30 s, and extension at 72 °C for 1 min 10 s, with a final extension at 72 °C for 5 min. For qRT-PCR targeting the ORF6 gene, and for primers targeting the Nsp2 gene and PRRSV whole genome, the primer sequences were as previously described [29].

### Detection and sequencing

RNA was extracted from samples using an RNA extraction kit (Vazyme, Nanjing, China) according to the manufacturer’s instructions. Subsequently, samples were subjected to detection by qRT-PCR targeting the ORF6 gene. Finally, RT-PCR amplification of the ORF5 gene was performed exclusively on samples with Ct values below 28. RT-PCR Amplification of ORF5 and Nsp2 genes from isolated PRRSV strains. RT-PCR products were sequenced (Tsingke, Beijing, China). Sequencing results were assembled using SeqMan (DNASTAR, Madison, WI, USA).

### Virus isolation

PAM cells were isolated from lung tissues of healthy 4-week-old piglets negative for both PRRSV antigen and antibody, and stored in liquid nitrogen. PRRSV-positive supernatants with Ct values below 28 were filtered through a 0.22 μm filter (EMD Millipore, Billerica, MA, USA) and inoculated into PAM cells. After 3 days of culture, the cells were subjected to three freeze-thaw cycles to harvest the viral fluid. The harvested viral fluid was then inoculated into fresh PAM cells. Virus isolation was confirmed by qRT-PCR. The isolated PRRSV was inoculated into Marc-145 cells. After 3 days of culture, the cells were subjected to three freeze-thaw cycles to harvest the viral fluid. The harvested viral fluid was continuously inoculated into Marc-145 cells. Virus isolation was confirmed by qRT-PCR. PAM and Marc-145 cells were cultured in RPMI 1640 and DMEM media (Gibco, Grand Island, NY, USA) supplemented with 10% fetal bovine serum (FBS) (Gibco, Grand Island, NY, USA), respectively.

### Indirect immunofluorescence (IFA)

The isolated PRRSV was inoculated into PAM or Marc-145 cells and incubated at 37 °C for 2 h, followed by culture in RPMI 1640 or DMEM containing 2% FBS, 100 U/mL penicillin, and 100 µg/mL streptomycin (Solarbio, Beijing, China). At 72 h post-infection, cells were washed with PBS and fixed with ethanol at room temperature for 15 min. After three washes with PBS, cells were blocked with 5% BSA (Biosharp, Beijing China) for 2 h. Following three washes with PBS, cells were incubated with PRRSV N protein monoclonal antibody (GeneTex, San Diego, CA, USA) for 1 h. After three washes with PBS, cells were incubated with FITC-conjugated goat anti-mouse IgG (Proteintech, Rosemont, IL, USA) for 1 h. Following three washes with PBS, cells were stained with DAPI (Beyotime, Shanghai, China) for 15 min and observed under a fluorescence microscope.

### Plaque assay

Marc-145 cell adapted PRRSV was diluted to 10^− 7^ and inoculated into Marc-145 cells, followed by incubation at 37 °C for 2 h. After three washes with PBS, DMEM containing 1% low-melting-point agarose, 2% FBS, 100 U/mL penicillin, and 100 µg/mL streptomycin was added. After cooling and solidification, the cells were cultured. At 72 h post-infection, the overlay agar was removed, and the cells were fixed with ethanol for 30 min. After three washes with PBS, cells were stained with 0.1% crystal violet for 10 min. Following three washes with PBS and drying, plaques were observed under an inverted fluorescence microscope.

### Whole genome sequencing

Viral RNA was extracted using an RNA extraction kit (Vazyme, Nanjing, China), and cDNA was synthesized using a reverse transcription kit (TaKaRa, Beijing, China). PCR amplification of the whole genome was performed using specific primers. The thermal cycling conditions were as follows: initial denaturation at 95 °C for 3 min, followed by 35 cycles of denaturation at 95 °C for 15 s, annealing at 54 °C for 15 s, and extension at 72 °C for 1 min, with a final extension at 72 °C for 3 min. PCR products were purified and cloned into the pMD-19T vector (TaKaRa, Beijing, China). Plasmids were recovered from positive colonies and sequenced (Tsingke, Beijing, China). Sequencing results were assembled using SeqMan (DNASTAR, Madison, WI, USA).

### Sequence alignment and phylogenetic analysis

Thirty-one PRRSV whole genome sequences were downloaded from the GenBank database for use as reference strains in bioinformatics analysis [29]. Sequences of the ORF5, Nsp2 genes, and whole genomes were aligned using MEGA 11, and phylogenetic trees were constructed using the neighbor-joining method with 1,000 bootstrap replicates [30]. Recombination analysis of PRRSV whole genomes was performed using Simplot and RDP4 software [14].

### Statistical analysis

Statistical analysis was performed using GraphPad Prism 8 (San Diego, USA). The chi-square test was used to determine significant differences between two groups. Significance levels were set at *p* < 0.05 (*), *p* < 0.01 (**), *p* < 0.001 (***)。The 95% confidence interval (CI) for the average positive detection rate was calculated using the formula: 95% CI = *p* ± 1.96 × √[p(1-p)/n].

## Results

### Detection of PRRSV2 in clinical samples and Phylogenetic analysis of the ORF5 gene

**Fig. 1:**
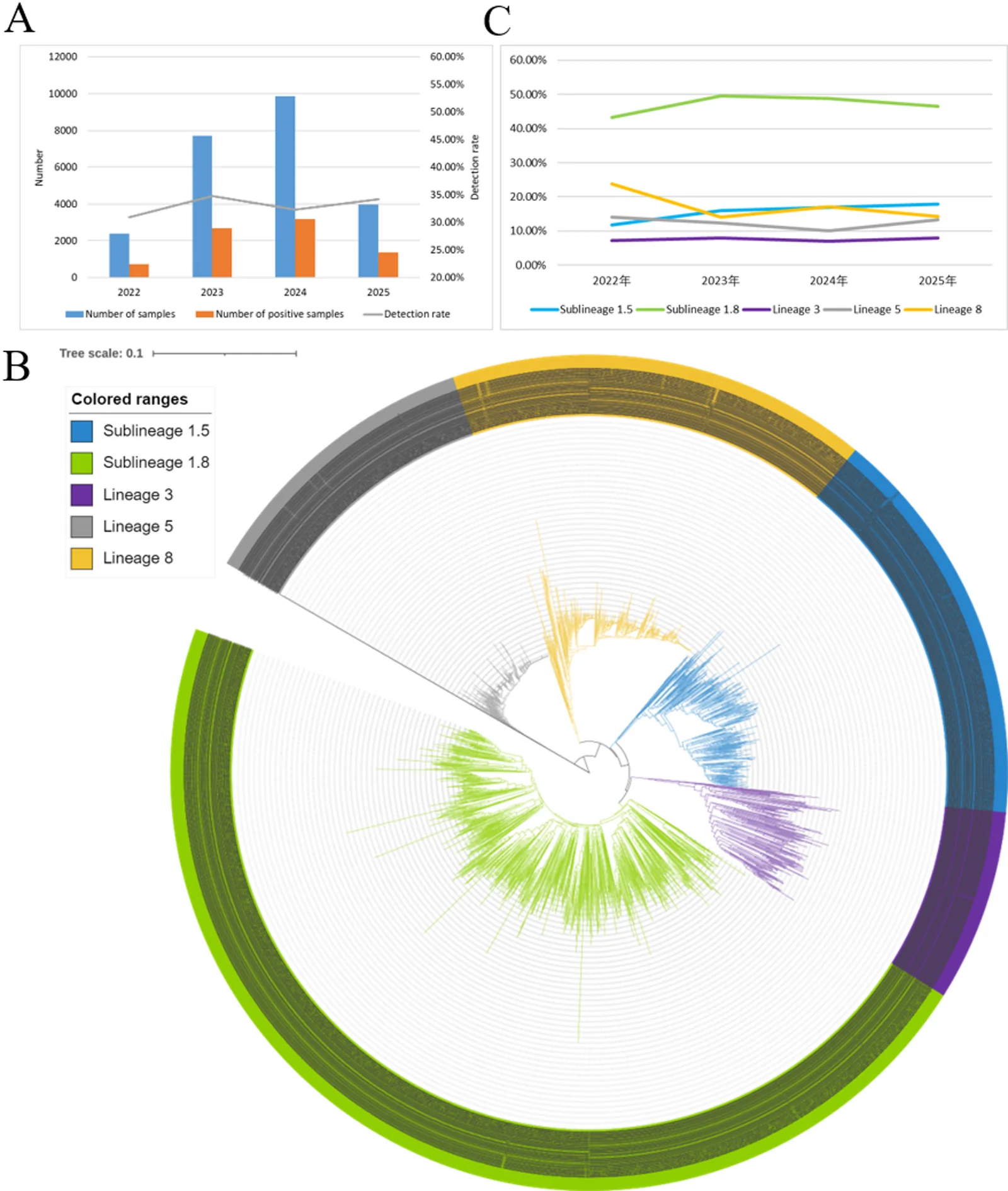
Average positive detection rate of PRRSV2 in clinical samples and phylogenetic analysis results of ORF5 gene from 2022 to 2025. (**A**) Average positive detection rate of PRRSV2 in clinical samples from 2022 to 2025. (**B**) Phylogenetic tree of 2,433 ORF5 gene sequences. (**C**) Lineage changes of ORF5 gene from 2022 to 2025. In A and C, blue indicates sublineage 1.5, green indicates sublineage 1.8, purple indicates lineage 3, gray indicates lineage 5, orange indicates lineage 8

Among the 23,962 samples collected from 2022 to 2025, 7,977 were positive, with an average positive detection rate of 33.29% (95% CI 32.69%-33.89%). The average annual positive detection rates were 30.89% (95% CI 29.04%-32.74%), 34.79% (95% CI 33.73%-35.85%), 32.34% (95% CI 31.42%-33.26%), and 34.17% (95% CI 32.70%-35.64%), respectively (Fig. 1A). These results indicate that PRRSV2 remains one of the major pathogens in pigs with abortion or respiratory symptoms in China. Phylogenetic analysis was performed using 31 ORF5 gene reference sequences from NCBI and 2,433 ORF5 gene sequences obtained from sequencing. The results showed that sublineage 1.8 (NADC30-like) accounted for 48.13% (1,171 sequences), sublineage 1.5 (NADC34-like) for 16.19% (394 sequences), lineage 3 (QYYZ-like) for 7.56% (184 sequences), lineage 5 (VR-2332-like) for 11.76% (286 sequences), and lineage 8 (CH-1a-like and JXA1-like) for 16.36% (398 sequences) (Fig. 1B). These findings indicate that sublineage 1.8 PRRSV is predominant in China, followed by sublineage 1.5 and lineage 8. From 2022 to 2025, the proportions of different lineages showed some changes: the proportions of sublineage 1.8, lineage 3, and lineage 5 remained relatively stable, while sublineage 1.5 increased from 11.74% to 17.82%, and lineage 8 decreased from 23.86% to 14.36% (Fig. 1C).

### Isolation of circulating PRRSV strains

**Fig. 2:**
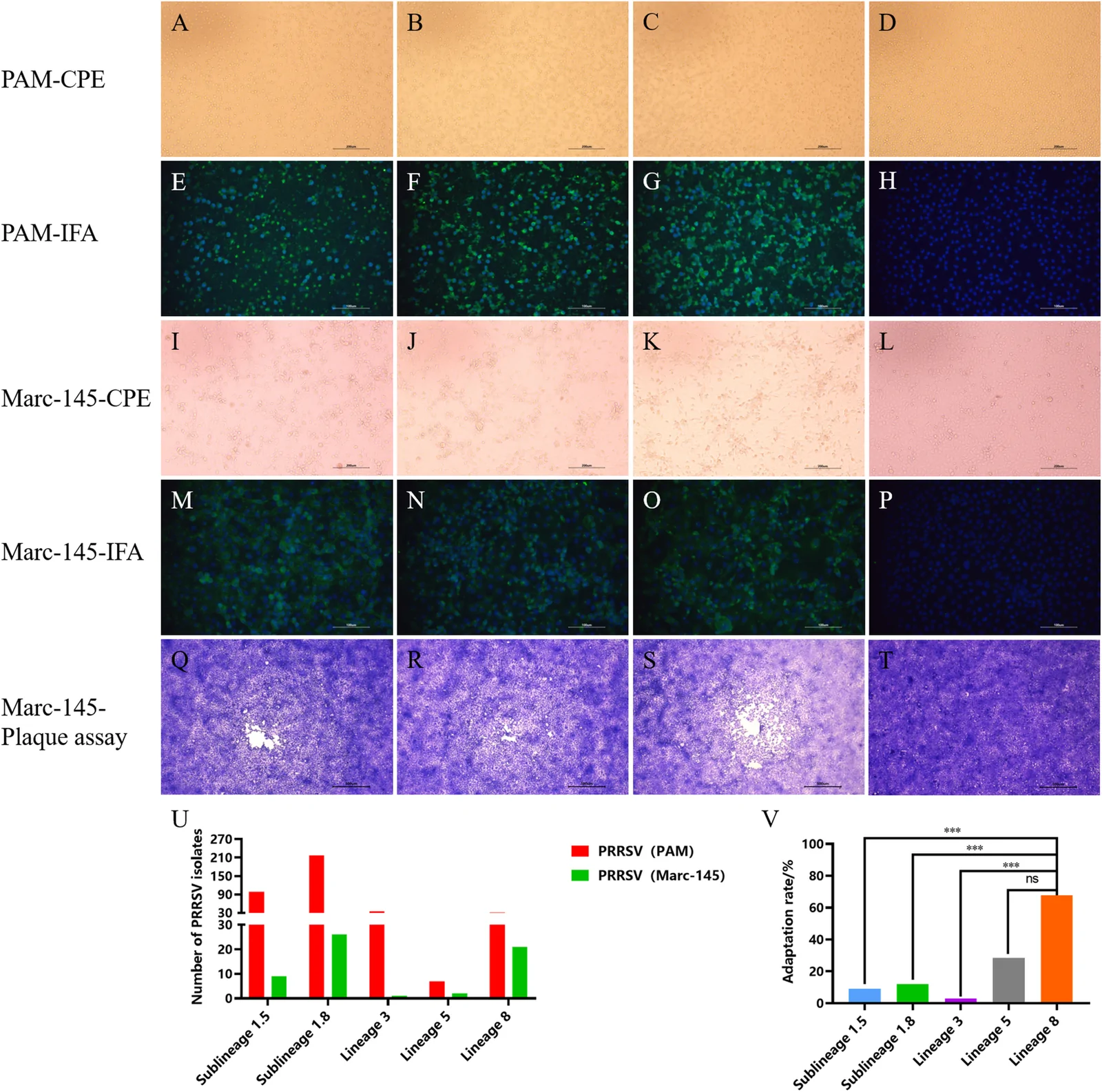
CPE, IFA, plaque assay, and virus isolation results of PRRSV isolates. PAM cells at 72 h post-inoculation with PRRSV (**A**-**C**, **E**-**G**). Uninoculated PAM cells (**D** and **H**). Marc-145 cells at 72 h post-inoculation with PRRSV (I-K, M-O). Uninoculated Marc-145 cells (**L** and **P**). Plaques formed by selected PRRSV isolates in Marc-145 cells (**Q**-**S**). Uninoculated Marc-145 cells (**T**). Number of PRRSV strains from different lineages adapted to PAM and Marc-145 cells (**U**). Adaptation rates of PRRSV strains from different lineages in Marc-145 cells (**V**). Asterisks (*) indicate significant differences in adaptation rates between sublineage 1.5, sublineage 1.8, and lineage 3 versus lineage 8 PRRSV in Marc-145 cells (*: *p* < 0.05; **: *p* < 0.01; ***: *p* < 0.001). Original magnification: 40× (Plaque assay), 100× (CPE), and 200× (IFA)

In this study, a total of 2,546 PRRSV positive samples with Ct values below 28 were collected. Virus isolation was performed using primary porcine alveolar macrophage (PAM) cells, resulting in the isolation of 389 PRRSV strains. The isolation period spanned from 2022 to 2025, covering 27 provincial-level administrative regions across China. A total of 6 NADC34-like strains were isolated from 4 provinces in China. These included the 3 previously reported strains (HeB-05, SD-02, and JL-1152) and 3 newly isolated strains (JL-1740-2024, HL-1885-2024, and JL-2380-2025). All isolated strains could stably proliferate and be passaged in PAM cells. Approximately 72 h post-inoculation, obvious cytopathic effects (CPE) were observed in PAM cells, characterized by cell shrinkage, fragmentation, death, and detachment. Green fluorescence was detected by IFA (Fig. 2A-H). All isolated PRRSV strains were subjected to adaptive passage in Marc-145 cells. The results showed that only 59 strains could adapt to Marc-145 cells (Fig. 2U). These 59 PRRSV strains caused obvious CPE in Marc-145 cells approximately 72 h post-inoculation. Green fluorescence was detected by IFA, and large, round, clear plaques were observed in plaque assays (Fig. 2I-T). The viral titers of 59 PRRSV strains in Marc-145 cells ranged from 10^4.20^ to 10^8.33^ TCID_50_/mL. The adaptation rates of different PRRSV lineages in Marc-145 cells varied (Fig. 2V). The average adaptation rate was 9.09% (9/99) for sublineage 1.5, 11.98% (26/217) for sublineage 1.8, 2.86% (1/35) for lineage 3, 28.57% (2/7) for lineage 5, and 67.74% (21/31) for lineage 8. The adaptation rates of lineage 1 and lineage 3 strains in Marc-145 cells were significantly lower than those of lineage 8 (*p* < 0.001), whereas no significant statistical difference was observed between lineage 5 and lineage 8 strains. Currently, the predominant circulating strains in China belong to lineage 1, and an increasing number of PRRSV isolates fail to adapt to Marc-145 cells. The results of this study are consistent with this trend.

### Phylogenetic analysis of ORF5 gene in circulating PRRSV strains

**Fig. 3:**
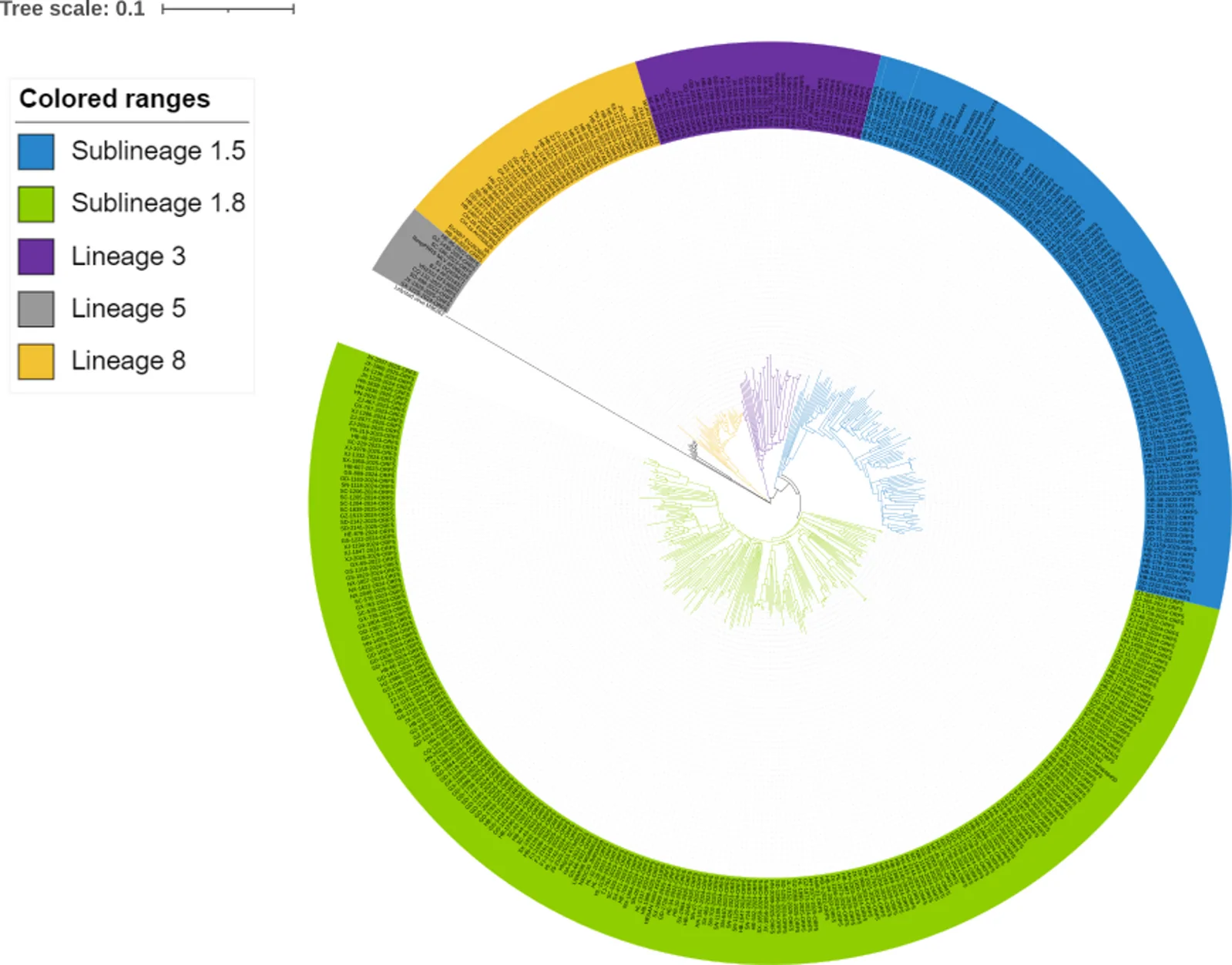
Phylogenetic tree of ORF5 gene in 389 PRRSV strains. Blue indicates sublineage 1.5, green indicates sublineage 1.8, purple indicates lineage 3, gray indicates lineage 5, orange indicates lineage 8

Phylogenetic analysis of the ORF5 gene of 389 PRRSV strains showed that sublineage 1.5 accounted for 25.45% (99 strains), sublineage 1.8 for 55.78% (217 strains), lineage 3 for 9.00% (35 strains), lineage 5 for 1.80% (7 strains), and lineage 8 for 7.97% (31 strains) (Fig. 3).

The nucleotide and amino acid identities of the ORF5 gene among the 389 PRRSV strains ranged from 78.1% to 100.0% and 75.1%-100.0%, respectively. The nucleotide identities with reference strains VR-2332, QYYZ, JXA1, IA/2014/NADC34, and NADC30 were 81.5%-99.2%, 80.7%-96.1%, 81.2%-99.5%, 82.6%-96.6%, and 81.8%-94.7%, respectively. The amino acid identities with these reference strains were 79.2%-98.6%, 78.3%-97.1%, 81.2%-98.6%, 81.6%-97.1%, and 82.1%-96.6%, respectively. The lowest nucleotide and amino acid identities were observed with reference strain Lelystad Virus, ranging from 57.5% to 62.5% and 50.7%-57.0%, respectively (Table 1).

**Table 1:** Nucleotide identities and amino acid similarities of ORF5 of 389 PRRSV strains

ORF5		Identity %							
		VR-2332	RespPRRS MLV	CH-1a	QYYZ	JXA1	IA/2014/ NADC34	NADC30	Lelystad Virus
389 PRRSV	nt	81.5–99.2	81.5–99.5	82.6–95.5	80.7–96.1	81.2–99.5	82.6–96.6	81.8–94.7	57.5–62.5
	aa	79.2–98.6	79.2–99.0	81.6–94.7	78.3–97.1	81.2–98.6	81.6–97.1	82.1–96.6	50.7–57.0
Sublineage 1.5	nt	84.1–88.4	83.4–88.4	83.9–90.2	80.7–87.0	83.9–90.8	85.7–96.6	85.8–90.8	59.1–62.3
	aa	81.2–87.9	82.1–87.9	83.1–89.9	80.2–88.4	83.6–89.9	87.9–97.1	86.0-93.7	51.2–56.5
Sublineage 1.8	nt	82.0-86.8	81.8–86.6	82.6–88.1	80.7–86.2	81.2–86.8	84.2–89.9	89.5–94.7	57.5–62.5
	aa	79.2–87.4	79.2–87.0	81.6–89.4	78.3–86.5	81.2–88.4	84.5–93.7	87.9–96.6	50.7–57.0
Lineage 3	nt	81.5–86.0	81.5–86.0	83.1–86.5	89.4–96.1	82.3–85.3	82.6–87.6	81.8–88.1	58.5–62.0
	aa	79.7–84.5	79.2–85.5	82.1–87.4	89.4–97.1	81.6–86.0	81.6–89.4	82.1–88.9	51.7–55.6
Lineage 5	nt	93.1–99.2	93.4–99.5	89.0-92.3	84.2–85.3	87.0-89.7	88.2–90.2	86.0–87.0	61.0-62.2
	aa	92.3–98.6	93.2–99.0	90.3–92.8	81.6–85.5	87.4–88.4	86.0-88.9	84.1–88.4	54.1–56.0
Lineage 8	nt	84.1–89.5	84.1–89.5	88.9–95.5	81.6–85.5	89.2–99.5	84.2–88.1	84.9–87.3	60.1–61.8
	aa	83.6–88.9	83.1–88.4	89.4–94.7	78.7–84.5	87.9–98.6	84.5–88.9	84.5–88.4	53.6–57.0

### Amino acid variation analysis of GP5 protein in circulating PRRSV strains

**Fig. 4:**
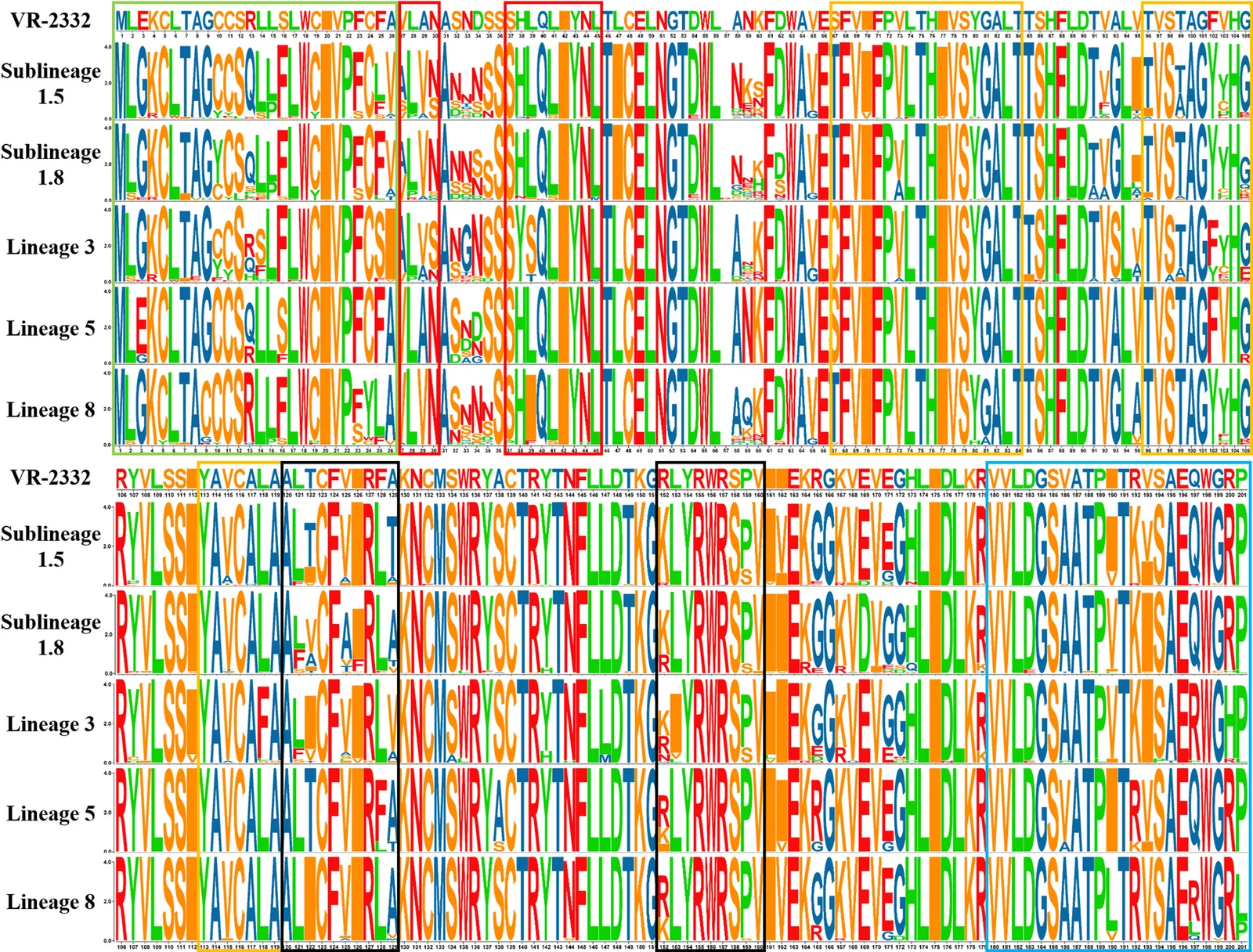
Amino acid variations, insertions, and deletions in the ORF5 protein of 389 PRRSV strains. The green box indicates the signal peptide; red boxes indicate epitopes A and B, orange boxes indicate three transmembrane regions, black boxes indicate two T-cell epitopes, blue boxes indicate B-cell epitopes. Eight sublineage 1.8 PRRSV strains had a 1 aa insertion at position 57. All PRRSV strains were aligned with the GP5 protein of VR-2332, comprising a total of 201 amino acid positions. Amino acid variations, deletions, and insertions were all based on the GP5 protein of VR-2332

Compared with the GP5 protein of reference strain VR-2332, the 389 PRRSV strains exhibited different amino acid deletions and insertions. 17.48% (68/389) of PRRSV strains showed a 1 aa deletion at position 32/35, including 4 sublineage 1.5 strains, 63 sublineage 1.8 strains, and 1 lineage 3 strain. Additionally, 8 sublineage 1.8 PRRSV strains exhibited a 1 aa insertion (A/K/R^57^) at position 57. Amino acid variations in the GP5 protein were mainly concentrated in the signal peptide, transmembrane region, and antigenic epitopes. Epitope A, located at positions 27–30 aa, exhibited amino acid variations including V^27^→A/S^27^, A^29^→V/T/G^29^, and N^30^→S/G^30^. Epitope B is a neutralizing antibody epitope located at positions 37–45 aa [7], amino acid variations mainly included H^38^→Y^38^ and L^39^→I/S/F/T^39^ (Fig. 4).

### Phylogenetic analysis of Nsp2 gene in circulating PRRSV strains

**Fig. 5:**
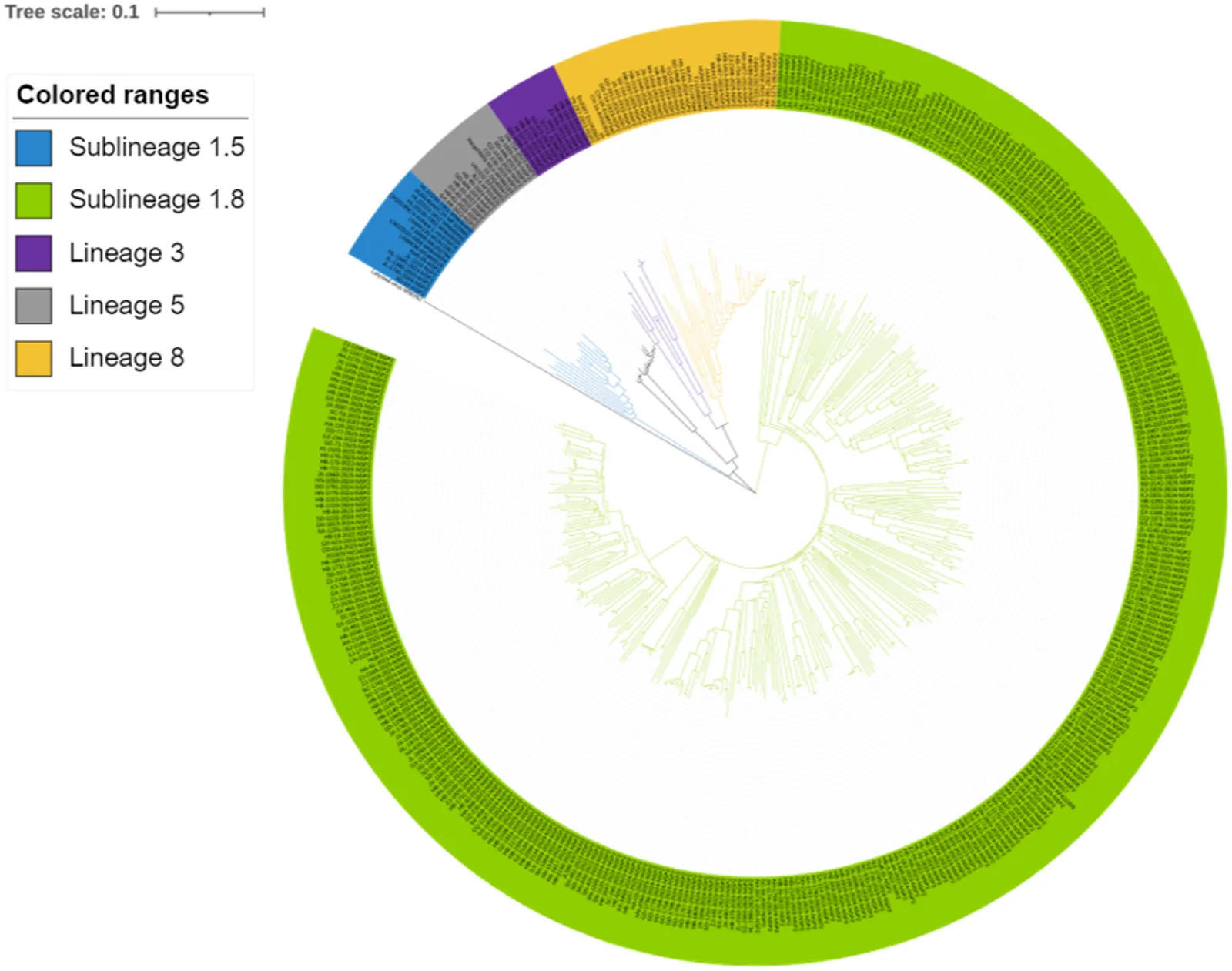
Phylogenetic tree of Nsp2 gene in 389 PRRSV strains. Blue indicates sublineage 1.5, green indicates sublineage 1.8, purple indicates lineage 3, gray indicates lineage 5, orange indicates lineage 8

Phylogenetic analysis of the Nsp2 gene of 389 PRRSV strains showed that lineage 1.5 accounted for 1.54% (6 strains), lineage 1.8 for 86.89% (338 strains), lineage 3 for 2.31% (9 strains), lineage 5 for 2.83% (11 strains), and lineage 8 for 6.43% (25 strains) (Fig. 5).

The nucleotide and amino acid identities of the Nsp2 gene among the 389 PRRSV strains ranged from 28.4% to 100.0% and 26.1%-100.0%, respectively. The nucleotide identities with reference strains VR-2332, QYYZ, JXA1, IA/2014/NADC34, and NADC30 were 42.7%-99.3%, 41.8%-86.9%, 37.0%-98.7%, 41.0%-95.2%, and 33.3%-95.9%, respectively. The amino acid identities with these reference strains were 41.8%-99.1%, 40.4%-79.6%, 38.7%-98.2%, 41.3%-92.1%, and 36.2%-93.3%, respectively. The lowest nucleotide and amino acid identities were observed with reference strain Lelystad Virus, ranging from 18.4% to 47.6% and 7.9%-38.7%, respectively (Table 2). The sequencing region of the Nsp2 gene in this study covered the hypervariable region, approximately 686 bp (NADC30-like) to 1,079 bp (VR-2332-like and CH-1a-like), resulting in relatively low homology with reference strains from different lineages.

**Table 2:** Nucleotide identities and amino acid similarities of Nsp2 of 389 PRRSV strains

Nsp2		Identity %							
		VR-2332	RespPRRS MLV	CH-1a	QYYZ	JXA1	IA/2014/ NADC34	NADC30	Lelystad Virus
389 PRRSV	nt	42.7–99.3	42.8–99.8	42.0-84.9	41.8–86.9	37.0-98.7	41.0-95.2	33.3–95.9	18.4–47.6
	aa	41.8–99.1	42.2–100.0	41.3–83.6	40.4–79.6	38.7–98.2	41.3–92.1	36.2–93.3	7.9–38.7
Sublineage 1.5	nt	48.4–56.9	48.3–57.0	51.1–60.6	49.0-56.2	45.7–55.0	82.2–95.2	72.9–74.6	37.9–39.6
	aa	46.1–51.9	45.8–51.9	49.7–56.9	48.1–52.6	46.1–53.0	78.2–92.1	69.9–71.2	27.9–36.9
Sublineage 1.8	nt	42.7–51.7	42.8–51.9	42.0-51.6	41.8–50.9	37.0-49.2	64.9–74.4	82.6–95.9	42.7–47.6
	aa	41.8–51.0	42.2–51.5	41.3–49.7	40.4–48.5	38.7–47.9	62.2–71.5	80.2–93.3	33.3–38.7
Lineage 3	nt	65.0-72.5	65.3–72.9	70.7–79.5	72.0-86.9	66.6–72.5	46.1–54.2	41.0-51.8	20.5–25.6
	aa	62.0-68.1	62.2–68.3	69.2–75.1	69.9–79.6	65.2–70.3	45.6–51.2	42.2–50.3	7.9–14.8
Lineage 5	nt	87.6–99.3	87.9–99.8	75.0-84.2	69.6–77.8	67.3–76.2	49.8–59.4	44.4–53.3	21.4–24.5
	aa	83.6–99.1	84.5–100.0	73.3–81.1	65.4–72.6	65.2–72.4	45.6–55.7	43.8–52.4	7.9–11.7
Lineage 8	nt	60.1–75.3	60.2–75.7	65.7–84.9	60.3–76.6	77.2–98.7	41.0-56.7	33.3–47.4	18.4–25.0
	aa	57.8–71.2	58.0-71.9	64.5–83.6	59.1–72.6	73.0-98.2	41.3–54.4	36.2–48.3	8.3–17.3

### Amino acid variation analysis of Nsp2 in circulating PRRSV strains

**Fig. 6:**
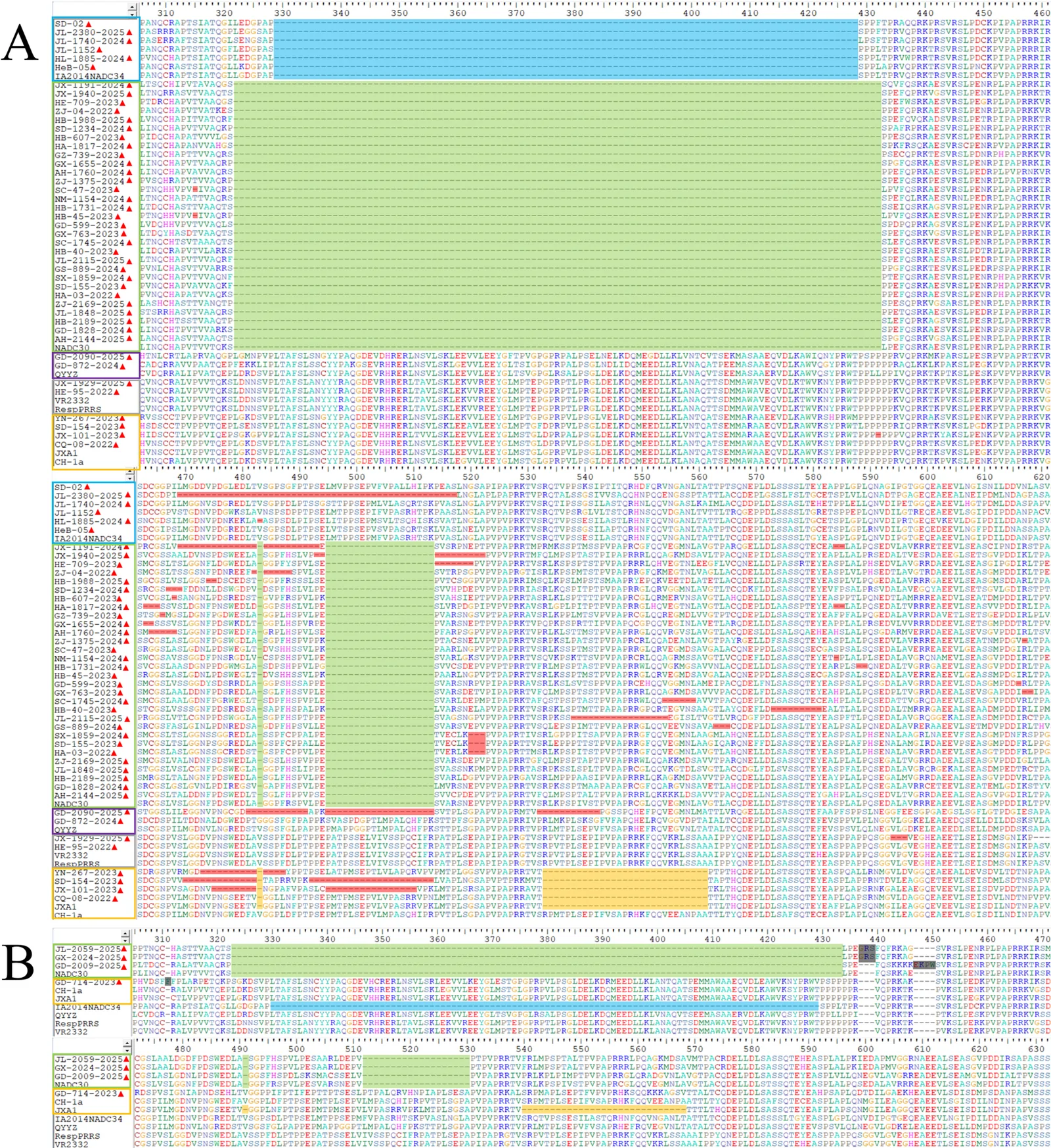
Schematic diagram of amino acid deletions and insertions in the Nsp2 protein of 48 representative PRRSV strains. (**A**) Amino acid deletions in Nsp2; (**B**) Amino acid insertions in Nsp2. Red triangles indicate PRRSV isolates; blue boxes indicate sublineage 1.5 PRRSV, green boxes indicate sublineage 1.8 PRRSV, purple boxes indicate lineage 3 PRRSV, gray boxes indicate lineage 5 PRRSV, orange boxes indicate lineage 8 PRRSV. Blue solid blocks indicate the 100 aa continuous deletion in NADC34-like PRRSV, green solid blocks indicate the 131 aa discontinuous deletion in NADC30-like PRRSV, orange solid blocks indicate the 30 aa discontinuous deletion in HP-PRRSV, red solid blocks indicate additional amino acid deletions based on the deletions of these strain types, gray solid blocks indicate amino acid insertions

Six sublineage 1.5 PRRSV strains were identified. Compared with the Nsp2 of VR-2332, all exhibited a consistent 100 aa continuous deletion identical to IA/2014/NADC34. Additionally, HL-1885-2024 showed an extra 1 aa deletion at position 483, while JL-2380-2025 exhibited an extra 49 aa deletion at positions 469–517. A total of 338 sublineage 1.8 PRRSV strains were identified. All strains exhibited a 131 aa discontinuous deletion consistent with NADC30. Additionally, 27.81% (94/338) of sublineage 1.8 PRRSV strains showed extra amino acid deletions, while 3 other strains had insertions of 3–4 aa. Nine lineage 3 PRRSV strains were identified. Five strains showed no amino acid deletions compared with VR-2332 and were consistent with QYYZ in this region, while the other 4 strains exhibited an additional 41 aa discontinuous deletion. Eleven lineage 5 PRRSV strains were identified. Ten strains were consistent with VR-2332, while 1 strain had an additional 3 aa deletion. Twenty-five lineage 8 PRRSV strains were identified. Twenty-four strains were consistent with JXA1, exhibiting a 30 aa discontinuous deletion. Among these, 3 strains had additional deletions of 14–35 aa based on the 30 aa deletion. The remaining 1 strain was consistent with VR-2332, with an additional 1 aa insertion (Fig. 6). A schematic diagram of amino acid deletions and insertions in the Nsp2 protein of 48 representative PRRSV strains is shown below:

### Phylogenetic analysis of whole genome of circulating PRRSV strains

**Fig. 7:**
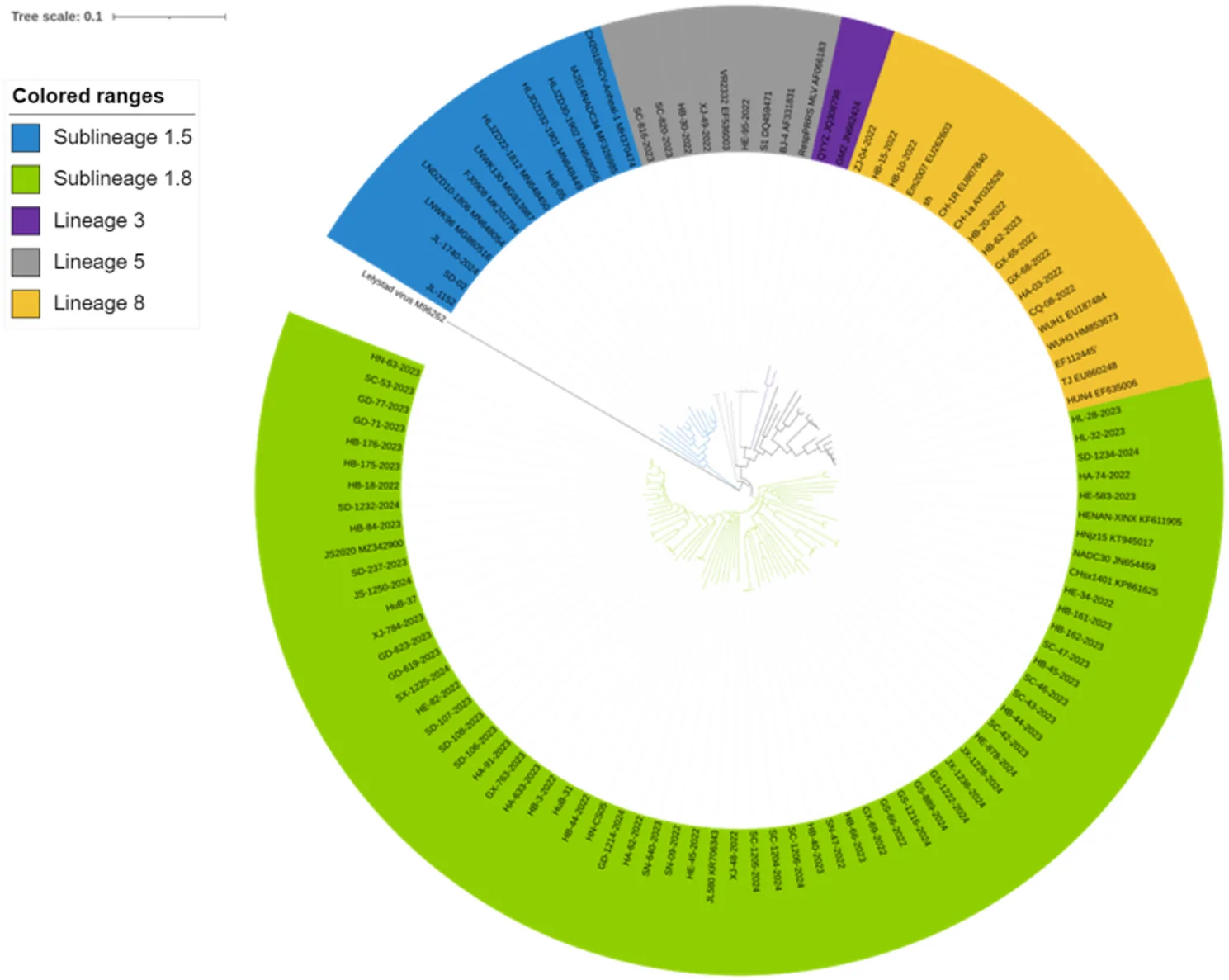
Phylogenetic tree of whole genome of 79 PRRSV strains. Blue indicates sublineage 1.5, green indicates sublineage 1.8, purple indicates lineage 3, gray indicates lineage 5, orange indicates lineage 8

Whole genome sequencing was performed on 79 PRRSV strains in this study, with genome lengths ranging from 14,980 to 15,519 bp. Phylogenetic analysis of the full-genome sequences showed that lineage 1.5 accounted for 5.06% (4 strains), lineage 1.8 for 77.22% (61 strains), lineage 5 for 6.33% (5 strains), and lineage 8 for 11.39% (9 strains) (Fig. 7).

### Recombination analysis of whole genome of circulating PRRSV strains

Recombination analysis of the 79 PRRSV whole genome sequences was performed using Simplot and RDP4, revealing that 93.67% (74/79) of strains exhibited inter-lineage recombination. The whole genome was divided into 16 segments based on 15 high-frequency recombination sites (Table 3). Segments 1–11 mainly included the 5’ UTR and non-structural protein regions from Nsp1α to Nsp12, while segments 12–16 mainly included structural protein regions from ORF2 to ORF7 and the 3’ UTR. Sequence alignment, phylogenetic tree construction, and genotyping were performed for these segments.

**Table 3:** 15 high-frequency recombination sites in the PRRSV whole genome

No.	Site/nt	No.	Site/nt	No.	Site/nt	No.	Site/nt	No.	Site/nt
1	687	4	3552	7	8021	10	11,552	13	13,202
2	1503	5	5388	8	10,703	11	12,099	14	13,886
3	2025	6	6420	9	11,144	12	12,663	15	14,518

Site based on PRRSV reference strain VR-2332

Based on phylogenetic analysis of the ORF5 gene, 79 whole-genome sequences were classified into different lineages. Sublineage 1.5 comprised 27 strains, of which 2 exhibited no detectable recombination and 25 displayed recombination events, with NADC34-like (2 strains) and NADC30-like (23 strains) as major parental strains. Sublineage 1.8 comprised 38 strains, of which 1 exhibited no detectable recombination and 37 displayed recombination events, with NADC30-like (35 strains) and JXA1-like (2 strains) as major parental strains. Lineage 3 comprised 5 strains, all of which were recombinant, with NADC30-like (4 strains) and JXA1-like (1 strain) as major parental strains. Lineage 5 comprised 2 strains, of which 1 exhibited no detectable recombination and 1 had NADC30-like as the major parental strain. Lineage 8 comprised 7 strains, of which 1 exhibited no detectable recombination and 6 were recombinant with JXA1-like as the major parental strain.

**Fig. 8:**
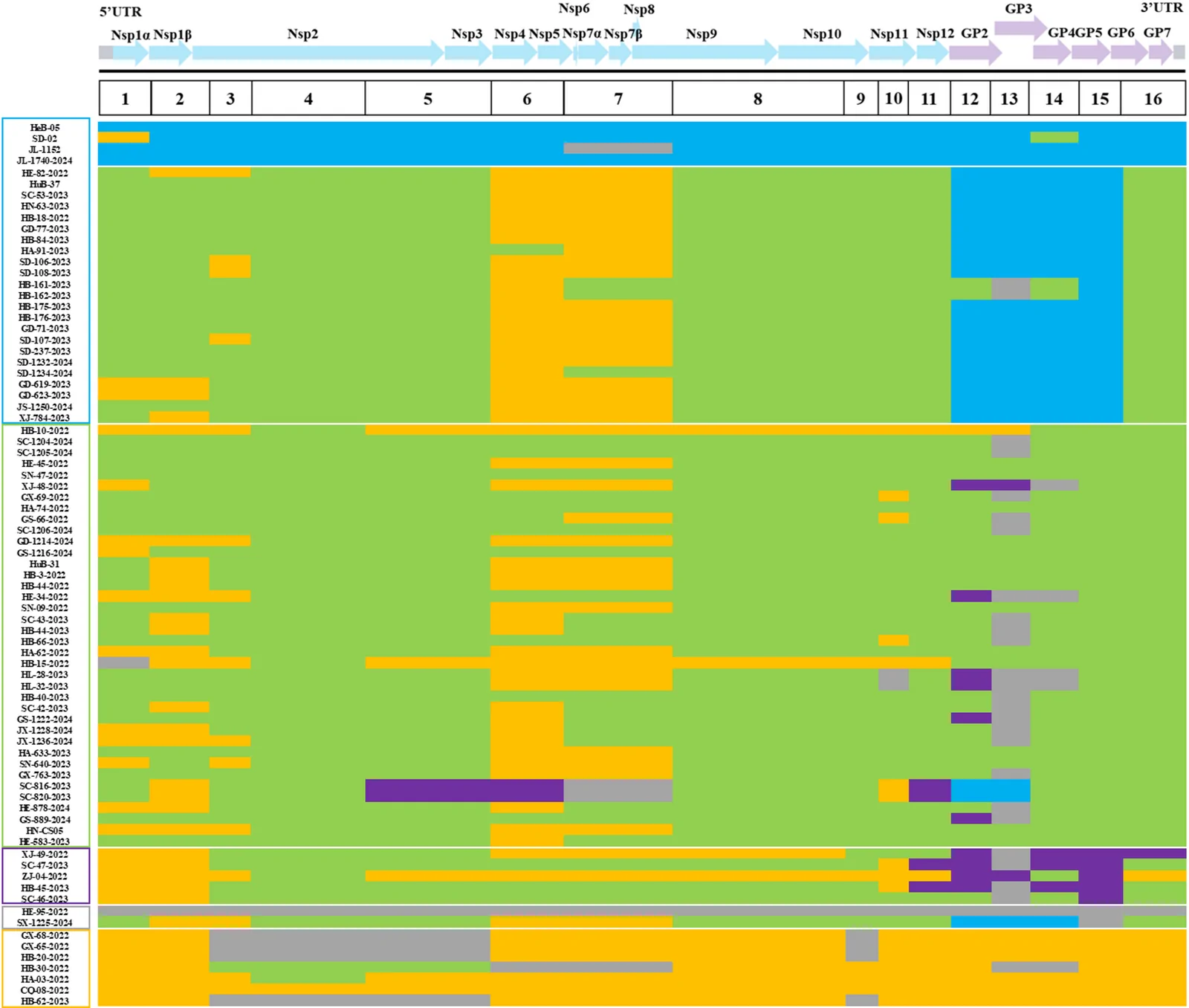
Recombination analysis of whole genome of 79 PRRSV strains. Blue boxes indicate sublineage 1.5 PRRSV; green boxes indicate sublineage 1.8 PRRSV; purple boxes indicate lineage 3 PRRSV; gray boxes indicate lineage 5 PRRSV; orange boxes indicate lineage 8 PRRSV. Blue solid blocks indicate sublineage 1.5 type; green solid blocks indicate sublineage 1.8 type; purple solid blocks indicate lineage 3 type; gray solid blocks indicate lineage 5 type; orange solid blocks indicate lineage 8 type

The major parental strains of 74 recombinant PRRSV strains and their respective proportions were NADC34-like (2.70%, 2/74), NADC30-like (85.14%, 63/74), and JXA1-like (12.16%, 9/74). The minor parental strains and their corresponding genomic regions included NADC34-like (ORF2-5), NADC30-like (Nsp2), JXA1-like (5’-Nsp1 and Nsp4-8), CH-1a-like (Nsp7), QYYZ-like (ORF2-5), and VR-2332-like (ORF3) (Fig. 8).

## Discussion

With the introduction and spread of sublineage 1.5 strains in recent years, the predominant circulating PRRSV strains in China have undergone some changes. Detection results showed that sublineage 1.8 remained the predominant circulating strain in China from 2022 to 2025, consistent with the situation before 2022. The average proportion of sublineage 1.5 nationwide increased steadily from 11.74% to 17.82%, becoming a secondary predominant strain together with lineage 8. Lineage 3 and lineage 5 strains continued to circulate at relatively stable levels without significant changes.

PAM cells are the most sensitive cells for PRRSV isolation; however, they are difficult to isolate and cannot be passaged [31]. Marc-145 cells can also be used for virus isolation; however, due to the prevalence of sublineage 1.5 and 1.8 PRRSV, a considerable number of strains cannot proliferate in Marc-145 cells [32–35]. In this study, 389 PRRSV strains were isolated using PAM cells, of which only 59 could proliferate in Marc-145 cells. Lineage 1 PRRSV showed lower adaptability in Marc-145 cells compared with lineage 8, which is consistent with current trends.

Deletions, insertions, and variations in the amino acid sequence of the PRRSV GP5 protein may lead to changes in viral replication and vaccine protection [4]. The GP5 proteins within different lineages showed high homology with their respective representative reference strains, with amino acid mutations mainly concentrated in the signal peptide, transmembrane region and antigenic epitope regions [36].

Nsp2 is the most variable region in the PRRSV genome, encompassing different types of amino acid deletions and insertions [8, 37]. In the alignment analysis of the Nsp2 hypervariable region of 389 PRRSV strains in this study, additional amino acid deletion sites were identified in sublineage 1.5, sublineage 1.8, lineage 3, lineage 5, and lineage 8 strains. Sublineage 1.8 strains exhibited the most amino acid changes, with 28.70% (97/338) of strains showing various types of additional amino acid deletions and insertions based on the 131 aa discontinuous deletion.

Phylogenetic analysis based on the ORF5 and Nsp2 genes of 389 PRRSV isolates revealed substantial discrepancies, suggesting the presence of numerous recombinant strains among these isolates. Recombination analysis of 79 whole-genome sequences confirmed this hypothesis. Recombinant PRRSV strains were predominantly sublineage 1.8 (70%, 2019–2023) and sublineage 8.7 (30%, 2019–2023) [38]. Among the 79 PRRSV strains subjected to whole genome sequencing in this study, 93.67% (74/79) exhibited inter-lineage recombination. Of the 74 recombinant strains, the major parental strains were sublineage 1.8 (85.14%, 63 strains), lineage 8 (12.16%, 9 strains), and sublineage 1.5 (2.70%, 2 strains). Compared with the study by You et al. (2019–2023), a new recombination type with sublineage 1.5 as the major parent was identified, the proportion of sublineage 1.8 as the major parent increased, and the proportion of lineage 8 as the major parent decreased. In the amino acid variations of the ORF5 and Nsp2 genes, sublineage 1.8 exhibited the most diverse variation types. In the whole genome recombination analysis, sublineage 1.8 also accounted for the highest proportion as the major parental strain, which may be associated with its rapid establishment as the predominant strain in China since its introduction in 2013. Whether the amino acid variations in ORF5 and Nsp2 and whole genome recombination are related to PRRSV pathogenicity requires further investigation.

Currently, sublineage 1.5 strains have become the secondary predominant strains in China, and continuous surveillance of these strains should be maintained. Meanwhile, two distinct recombination patterns were observed in the whole genome of sublineage 1.5 strains: one with NADC34-like as the major parental strain, exhibiting recombination with other strain types in limited genomic regions; and another with NADC30-like as the major parental strain, with the ORF2-5 region derived from NADC34-like. Updated vaccine strains should be developed to address these sublineage 1.5 strains with different recombination patterns.

## Conclusion

The average positive detection rate of PRRSV2 in pigs with abortion or respiratory symptoms in China from 2022 to 2025 was 33.29%. Sublineage 1.8 PRRSV remained predominant in China, followed by sublineage 1.5 and lineage 8. Extensive amino acid variations, insertions, and deletions were identified in the ORF5 and Nsp2 genes of 389 PRRSV strains. Among 79 PRRSV strains subjected to whole genome sequencing, 93.67% exhibited inter-lineage recombination, indicating the genetic diversity and complexity of PRRSV. Overall, this study demonstrates the positive detection rate and predominant strain types of PRRSV2 in China. Sequencing results of isolated PRRSV strains reveals amino acid variations and recombination in the ORF5, Nsp2, and whole genomes. This study provides data support for the molecular epidemiology of PRRSV2 in China.

## Data Availability

The datasets used and/or analysed during the current study are available from the corresponding author on reasonable request.
